# METRNL attenuates lipid-induced inflammation and insulin resistance via AMPK or PPARδ-dependent pathways in skeletal muscle of mice

**DOI:** 10.1038/s12276-018-0147-5

**Published:** 2018-09-13

**Authors:** Tae Woo Jung, Sung Hoon Lee, Hyoung-Chun Kim, Joon Seok Bang, A. M. Abd El-Aty, Ahmet Hacımüftüoğlu, Yong Kyoo Shin, Ji Hoon Jeong

**Affiliations:** 1 Research Administration Team, Seoul National University Bundang Hospital, Seongnam, Korea; 2 College of Pharmacy, Chung-Ang University, Seoul, Korea; 3 Neuropsychopharmacology and Toxicology Program, College of Pharmacy, Kangwon National University, Chunchon, Korea; 4 College of Pharmacy, Sookmyung Women’s University, Seoul, Korea; 5 Department of Pharmacology, Faculty of Veterinary Medicine, Cairo University, 12211 Giza, Egypt; 6 Department of Medical Pharmacology, Medical Faculty, Ataturk University, Erzurum, Turkey; 7 Department of Pharmacology, College of Medicine, Chung-Ang University, 221, Heuksuk-dong, Dongjak-gu, Seoul, 156–756 Korea

## Abstract

Physical activity has many beneficial effects on metabolic disorders, such as obesity, insulin resistance, and diabetes. Meteorin-like protein (METRNL), a novel secreted protein homologous to the neurotrophin Metrn, is induced after exercise in the skeletal muscle. Herein, we investigated the effects of METRNL on lipid-mediated inflammation and insulin resistance in skeletal muscle via AMP-activated protein kinase (AMPK) or peroxisome proliferator-activated receptor δ (PPARδ). Treatment with METRNL suppressed inflammatory markers, such as nuclear factor κB (NFκB) nuclear translocation, inhibitory κBα (IκBα) phosphorylation, interleukin-6 (IL-6) expression, and pro-inflammatory cytokines (such as TNFα and MCP-1). METRNL treatment also attenuated the impaired insulin response both in palmitate-treated differentiated C2C12 cells and the skeletal muscle of high-fat diet (HFD)-fed mice. Furthermore, METRNL administration rescued glucose intolerance and reduced HFD-induced body weight gain in mice; however, METRNL did not affect calorie intake. METRNL treatment increased AMPK phosphorylation and PPARδ expression both in differentiated C2C12 cells and mouse skeletal muscle. siRNA-mediated suppression of AMPK and PPARδ abrogated the suppressive effects of METRNL on palmitate-induced inflammation and insulin resistance. Moreover, METRNL augmented the mRNA expression of fatty acid oxidation-associated genes, such as carnitine palmitoyltransferase 1 (CPT1), acyl-CoA oxidase (ACO), and fatty acid binding protein 3 (FABP3). siRNAs for AMPK and PPARδ reversed these changes. In the current study, we report for the first time that METRNL alleviates inflammation and insulin resistance and induces fatty acid oxidation through AMPK or PPARδ-dependent signaling in skeletal muscle.

## Metabolic disorders: A hormone treatment for diabetes and obesity

Treatment with a hormone that is normally induced following exercise helps alleviate inflammation and insulin resistance in the muscles of obese mice, highlighting a potential new therapeutic strategy for diabetes and other metabolic disorders. Meteorin-like protein (METRNL) is a hormone produced by muscle cells after physical activity and on exposure to cold. A team led by Ji Hoon Jeong and Yong Kyoo Shin from Chung-Ang University in Seoul, South Korea, observed that levels of METRNL are reduced in mice fed a high-fat diet and in mouse skeletal muscle cells chemically treated to become unresponsive to insulin. Administering METRNL to the cells or mice reduced levels of inflammation, resulting in improved insulin responses. However, these improvements occurred only when certain regulatory molecules were active, revealing a key pathway through which METRNL acts.

## Introduction

Although regular physical activity appears to have beneficial effects on insulin sensitivity in humans, the potential mechanisms underlying these effects remain to be elucidated^1^. Myokines, such as fibroblast growth factor 21 (FGF21), irisin, β-aminoisobutyric acid (BAIBA), and meteorin-like protein (METRNL), may partly mediate the beneficial effects of regular exercise on whole-body function^2^. METRNL, also known as subfatin, is a circulating protein expressed in monocytes, adipocytes, and skeletal muscle^3,4^. METRNL is induced in skeletal muscle upon exercise^5^ and in white adipose tissue upon cold exposure^6^. METRNL elevates whole-body energy expenditure and improves glucose tolerance by enhancing the browning of white adipose tissue via a STAT6-mediated pathway in adipose tissue infiltrated by macrophages^6^. Furthermore, METRNL transgenic mice have shown that METRNL improves insulin sensitivity through activation of PPARγ-mediated signaling, which was assumed to play a crucial role in the regulation of adipocyte differentiation^5,7^. However, the direct effects of METRNL on inflammation and insulin signaling in skeletal muscle and its underlying mechanisms remain unknown.

Increased serum-free fatty acid levels are detected in patients with insulin resistance^8^. Saturated free fatty acid causes impaired insulin signaling^9^ and inflammation, leading to insulin resistance through various pathways associated with diacylglycerol-mediated protein kinase C^10^, Toll-like receptor (TLR)-2^11^, or TLR-4^12^. These pathways activate nuclear factor κB (NFκB), a well-known pro-inflammatory transcription factor that induces insulin resistance in skeletal muscle^13^. Activation of the NFκB-mediated pathway stimulates the expression of pro-inflammatory cytokines, such as TNFα and IL-6, which play a crucial role in the development of insulin resistance and type 2 diabetes^14^. Therefore, proper suppression of lipid-induced inflammation is of critical importance for the treatment of diabetes.

In the present study, we investigated the effects of the novel myokine, METRNL, on lipid-induced inflammation and insulin resistance. Additionally, we explored a downstream pathway related to AMP-activated protein kinase (AMPK) and peroxisome proliferator-activated receptor δ (PPARδ) in differentiated C2C12 cells and mouse skeletal muscle.

## Materials and methods

### Cell cultures, reagents, and antibodies

The mouse skeletal muscle cell line C2C12 (ATCC, Manassas, VA, USA) was cultured in Dulbecco’s modified eagle medium (DMEM; Invitrogen, Carlsbad, CA, USA) supplemented with 10% fetal bovine serum (Invitrogen), 100 units/mL penicillin, and 100 μg/mL streptomycin (Invitrogen). Cells were cultured and maintained at 37 °C in a humidified atmosphere of 5% CO_2_. Cells were supplemented with 2% horse serum (for 48 h) to induce differentiation. C2C12 cells were confirmed to be free from mycoplasma. We used cells at passages 5–10 for all experiments. Mouse recombinant METRNL (Adipogen, San Diego, CA, USA) was dissolved in phosphate-buffered saline (PBS). Sodium palmitate (Sigma, St Louis, MO, USA) was conjugated to 2% BSA (fatty acid free grade; Sigma) dissolved in DMEM. In all experiments, cells were treated with palmitate-BSA for 24 h, and 2% BSA was used as a control. Cells were treated with 0–200 ng/mL METRNL^7^ and 200 μM palmitate for 24 h without palmitate pretreatment or additional treatment steps. Cells were cultured in serum starvation media (without FBS) for 6 h before insulin treatment. Insulin (10 nM) was used to stimulate insulin signaling, IRS-1 and Akt for 3 min after METRNL and palmitate treatment.

### Animals, feeding, and treatment

This study was approved by the Institutional Animal Review Board of Chung Ang University, Seoul, Republic of Korea. Animal studies were conducted in accordance with the *Guide for the Care and Use of Laboratory Animals* (NIH publication, 8th edition, 2011).

#### Experiment 1

A control and two experimental groups of 8-week-old male C57BL/6J (B6) mice were given a normal diet (ND; Brogaarden, Gentofte, Denmark) or a high-fat diet (HFD; Research Diets, New Brunswick, NJ, USA) for 8 weeks. The HFD plus METRNL group was additionally administered METRNL intravenously (2 μg/mouse/day)^7^, and the ND and HFD groups were administered vehicle intravenously at the same volume (mouse/day) for 8 weeks. Mouse soleus skeletal muscle samples were isolated 10 min after intraperitoneal injection of insulin (Novo Nordisk, Princeton, NJ, USA; 10 U/kg body weight). The intraperitoneal glucose tolerance test (IPGTT) was performed as follows: mice were fasted for 12 h (overnight) and then intraperitoneally injected with glucose (2 g/kg body weight). Thereafter, serum glucose levels were measured at 0, 30, 60, 90, and 120 min post administration. To perform the insulin tolerance test (ITT), mice were fasted for 6 h and then given an intraperitoneal injection of human insulin (1 U/kg body weight). Serum glucose levels were measured at 0, 15, 30, 45, and 60 min thereafter. The IPGTT was performed 3 days before the mice treated with HFD and METRNL for 8 weeks were sacrificed. One day after the end of the IPGTT, the ITT was performed. Serum glucose levels were measured using an Accu-Check III glucose analyzer.

#### Experiment 2

A control and two experimental groups of 8-week-old male C57BL/6J (B6) mice were given a normal diet (ND; Brogaarden, Gentofte, Denmark) or a high-fat diet (HFD; Research Diets, New Brunswick, NJ, USA) for 2 weeks. The HFD plus METRNL group was additionally administered METRNL intravenously (2 μg/mouse/day), and the ND and HFD groups were administered vehicle intravenously at the same volume (mouse/day) for 2 weeks. AMPK siRNA (100 μg/mouse) or PPARδ siRNA (80 μg/mouse) was injected twice (at 48-h intervals) for 2 weeks into the HFD-fed and METRNL-treated AMPK or PPARδ-knockdown groups through the tail vein using in vivo-jetPEI^TM^ (Polyplus Transfection, NY, USA) for siRNA delivery into skeletal muscle. Scrambled siRNA was injected twice into each mouse in the ND group. Upon completion of the study period, all experimental mice were sacrificed under anesthesia after fasting overnight for 12 h.

### RNA extraction and quantitative real-time PCR

Total RNA from harvested C2C12 cells and soleus skeletal muscle tissue was isolated using TRIzol reagent (Invitrogen, Carlsbad, CA). Gene expression was measured by quantitative real-time PCR (qPCR) using the fluorescent TaqMan 5´ nuclease assay on an Applied Biosystems 7000 sequence detection system (Foster City, CA, USA). qPCR was performed using cDNA with 2× TaqMan Master Mix and the 20× premade TaqMan gene expression assays (Applied Biosystems). qPCR conditions were 95 °C for 10 min, followed by 40 cycles of 95 °C for 15 s and 60 °C for 1 min. Expression of mouse carnitine palmitoyltransferase 1 (*CPT1*) (Mm00463960_ml; Applied Biosystems), acyl-CoA oxidase (*ACO*) (Mm00801417_ml), fatty acid binding protein 3 (*FABP3*) (Mm02342495_ml), and spliced XBP-1 mRNA was normalized to the expression of mouse beta-actin (Mm00607939_sl; Applied Biosystems). Mouse spliced XBP-1 primers (forward: 5′-GAGTCCGCAGCAGGTG-3′ and reverse: 5′-GTGTCAGAGTCCATGGGA-3′) were used.

### Western blot analysis

Differentiated C2C12 cells were harvested, and proteins were extracted with lysis buffer (PRO-PREP; Intron Biotechnology, Seoul, Republic of Korea) for 60 min at 4 °C. Protein samples (30 μg) were subjected to 12% SDS-PAGE, transferred to a nitrocellulose membrane (Amersham Bioscience, Westborough, MA, USA), and probed with the indicated primary antibody followed by a secondary antibody conjugated with horseradish peroxidase (Santa Cruz Biotechnology). The signals were detected using enhanced chemoluminescence (ECL) kits (Amersham Bioscience). Anti-insulin receptor substrate (IRS)-1 (1:2500), anti-phospho-Akt (Ser473; 1:1000), anti-Akt (1:1000), anti-phospho-AMPK (Thr172; 1:1000), anti-AMPK (1:2500), anti-NFκBp65 (1:2500), anti-phospho-IκBα (Ser32; 1:1000), anti-phospho-eIF2α (Ser51; 1:1000), anti-eIF2α (1:1000), anti-PPARδ (1:2500), anti-PPARα (1:1000), anti-PPARγ (1:1000), anti-phospho-LKB1 (Ser428; 1:1000), anti-LKB1 (1:2500), anti-phospho-acetyl-CoA carboxylase (ACC) (Ser79; 1:1000), anti-ACC (1:2500), and anti-PGC1α (1:1000) antibodies were procured from Cell Signaling Technology (Beverly, MA, USA). Anti-phospho-IRS-1 (Tyr632; 1:1000) and anti-beta-actin (1:5000) antibodies were obtained from Santa Cruz Biotechnology (Santa Cruz, CA, USA). The anti-METRNL antibody (1:1000) was supplied by Abcam (Cambridge, MA, USA).

### Enzyme-linked immunosorbent assay (ELISA)

The concentrations of METRNL, TNFα, and MCP-1 in mouse serum were each measured using an ELISA kit (R&D Systems, Minneapolis, MN, USA) according to the manufacturer’s instructions.

### Transfection with siRNAs for gene silencing

Small interfering (si) RNA oligonucleotides (20 nM) specific for AMPK and PPARδ were acquired from Santa Cruz Biotechnology. To suppress gene expression, cell transfection was performed with Lipofectamine 2000 (Invitrogen) in accordance with the manufacturer’s instructions. In brief, the cells were grown to 50–60% confluence and fully differentiated, followed by serum starvation for 6 h. The cells were transfected with validated siRNA or scramble siRNA at a final concentration of 20 nM in the presence of the transfection reagent. After transfection, the cells were harvested at 36 h for protein extraction or further treated with 10 nM insulin, 20 μg/mL METRNL, or 200 μM palmitate.

### Cell fractionation for the measurement of NFκB nuclear translocation

Cells were fractionated using a Nuclear/Cytosol Fractionation Kit according to the manufacturer’s protocols (Bioviosion, Milpitas, CA, USA).

### Measurement of glucose uptake, acetyl-CoA, and ATP content

Glucose uptake levels were measured using the Glucose Uptake Assay Kit^TM^ (Abcam). In brief, proliferating C2C12 cells were seeded at 5 × 10^4^ cells/well in black-walled/clear-bottom 96-well plates (Corning, Inc., Corning, NY, USA) in DMEM containing 10% FBS. Upon reaching a confluency of 50%, differentiation was induced with differentiating media consisting of high-glucose DMEM and 2% horse serum. After 48 h, the media was changed to media containing palmitate (250 μM) or METRNL (0–200 ng/mL) for 24 h. Following treatment, the media was removed from the wells, and the cells were treated with 10 nM insulin and 1 mM 2-deoxyglucose (2-DG) for 30 min. Afterward, the plates were centrifuged for 1 min at 500 rpm and incubated for 1 h at room temperature. After 2-DG was taken up by the cells, it was extracted by extraction buffer in the kit. 2-DG uptake levels were measured at a wavelength of OD 412 nm on a BioTek Synergy HT plate reader (BioTek Instruments, Inc., Winooski, VT, USA). Intracellular levels of acetyl-CoA were measured using the PicoProbe Acetyl CoA Assay Kit^TM^ (Abcam), and intracellular ATP levels were measured using ATP Assay Kit^TM^ (Abcam) in differentiated C2C12 cells or soleus skeletal muscle according to the manufacturer’s instructions.

### Statistical analysis

All statistical analyses were conducted using the SPSS/PC statistical program (version 12.0 for Windows; SPSS, Chicago, IL, USA). The results are presented as the fold change of the highest value (mean ± SEM). All of the in vitro experiments were performed at least three times. All of the in vivo experiments were performed with five (experiment 1) or three (experiment 2) mice in each group. One-way ANOVA with Tukey’s post hoc test was used for statistical analysis.

## Results

### METRNL prevents impairment of insulin sensitivity in palmitate-treated C2C12 cells and skeletal muscle of HFD-fed mice

**Fig. 1:**
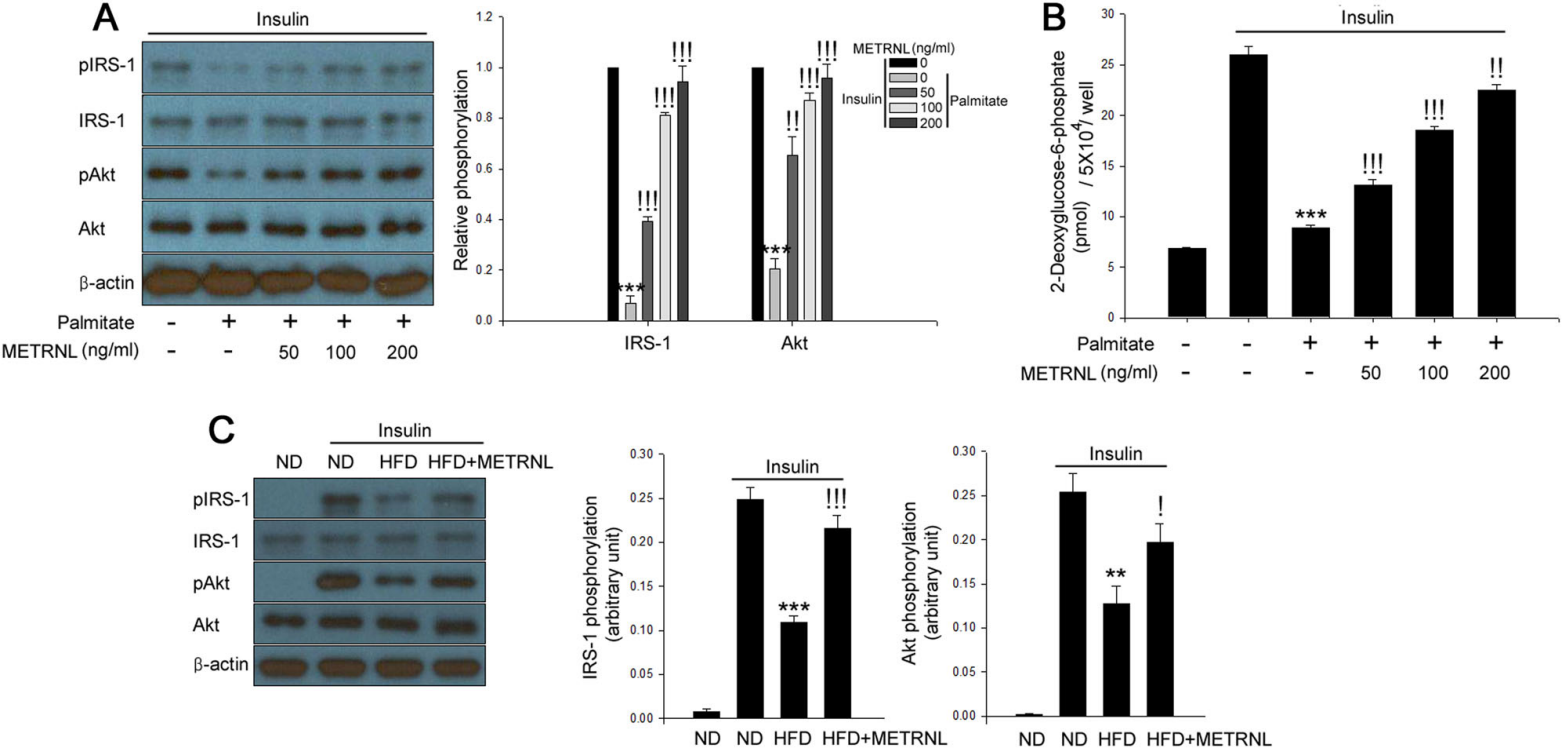
METRNL attenuates insulin resistance in differentiated C2C12 myocytes and soleus skeletal muscle of mice. **a** Western blot analysis of phosphorylation of IRS-1 and Akt and **b** 2-deoxyglucose uptake in differentiated C2C12 cells treated with palmitate (200 μM) and METRNL (0–200 ng/mL) for 24 h. Human insulin (10 nM) stimulates IRS-1 and Akt phosphorylation for 3 min. **c** Western blot analysis of IRS-1 and Akt phosphorylation in soleus skeletal muscle of HFD-fed mice treated with METRNL (5 animals/treatment group). Means ± SEM were calculated from three independent experiments. ^***^P < 0.001 and ^**^P < 0.01 compared to levels in the control or insulin-injected ND-fed groups.^!!!^*P* < 0.001,^!!^*P* < 0.01, and ^!^*P* < 0.05 compared to palmitate or insulin-injected HFD-fed groups

We first evaluated METRNL expression in palmitate-treated C2C12 cells and soleus skeletal muscle of HFD-fed mice to reveal its relevance under hyperlipidemic conditions. In agreement with a recent report^15^, HFD treatment decreased METRNL expression in skeletal muscle and its circulating levels in mice. Furthermore, treatment of C2C12 myocytes with palmitate significantly suppressed METRNL expression (Suppl. Fig. 1). Afterward, we selected and optimized the appropriate concentrations of METRNL^7^. To investigate the effects of METRNL on palmitate-induced insulin resistance, we examined the effect of METRNL on insulin-stimulated IRS-1 and Akt phosphorylation and glucose uptake. Differentiated C2C12 cells treated with palmitate and soleus skeletal muscle of HFD-fed mice exhibited an impaired insulin response. However, METRNL treatment markedly restored these changes in both C2C12 cells (Fig. 1a, b) and isolated soleus skeletal muscle of HFD-fed mice (Fig. 1c).

### METRNL attenuates HFD-induced insulin resistance in mice

**Fig. 2:**
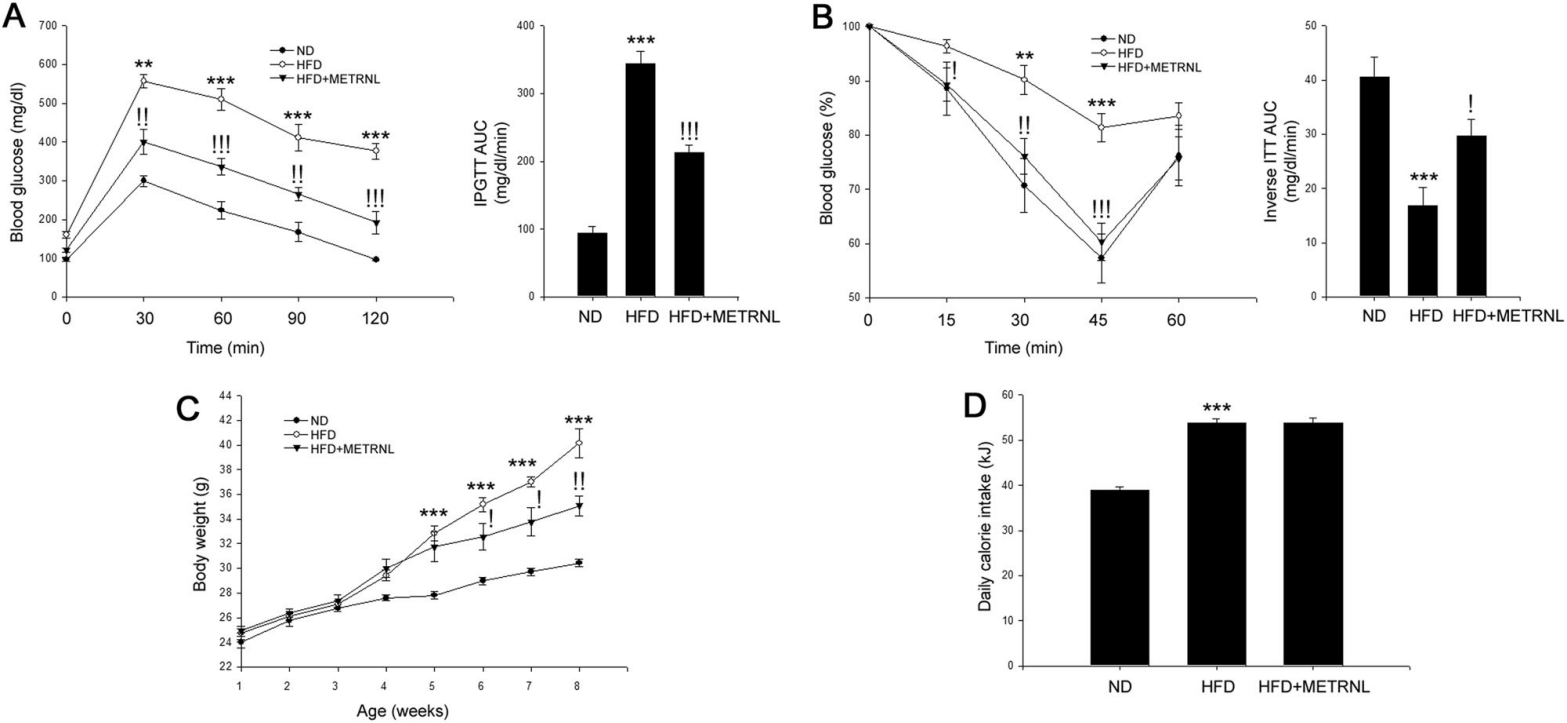
METRNL administration attenuates insulin resistance in mice. **a** The IPGTT of experimental mice and the glucose area under the curve (AUC) during the IPGTT. **b** The ITT and the glucose inverse AUC during the ITT. **c** Body weight measurement and **d** daily energy intake in mice (5 animals/treatment group). Closed circles, ND; opened circles, HFD; closed triangles, HFD + METRNL. Means ± SEM were calculated from five separate animals. ^***^P < 0.001 and ^**^P < 0.01 compared to ND treatment.^!!!^*P* < 0.001,^!!^*P* < 0.01 and ^!^*P* < 0.05 compared to HFD treatment

We next investigated the effects of METRNL on glucose tolerance and insulin sensitivity by performing the IPGTT and ITT. These tests revealed that HFD markedly aggravated glucose tolerance and decreased insulin sensitivity compared with ND. However, METRNL administration ameliorated HFD-induced glucose tolerance and insulin resistance (Fig. 2a, b). As the HFD-induced basal serum glucose levels were decreased by METRNL treatment; the ITT results were difficult to interpret. Therefore, we normalized the results to basal glycemia to investigate the effects of METRNL on insulin sensitivity (Fig. 2b). Moreover, METRNL treatment decreased HFD-induced body weight gain (Fig. 2c) but had no effect on calorie intake (Fig. 2d).

### METRNL ameliorates palmitate-induced inflammation in C2C12 myocytes and soleus skeletal muscle of HFD-fed mice

**Fig. 3:**
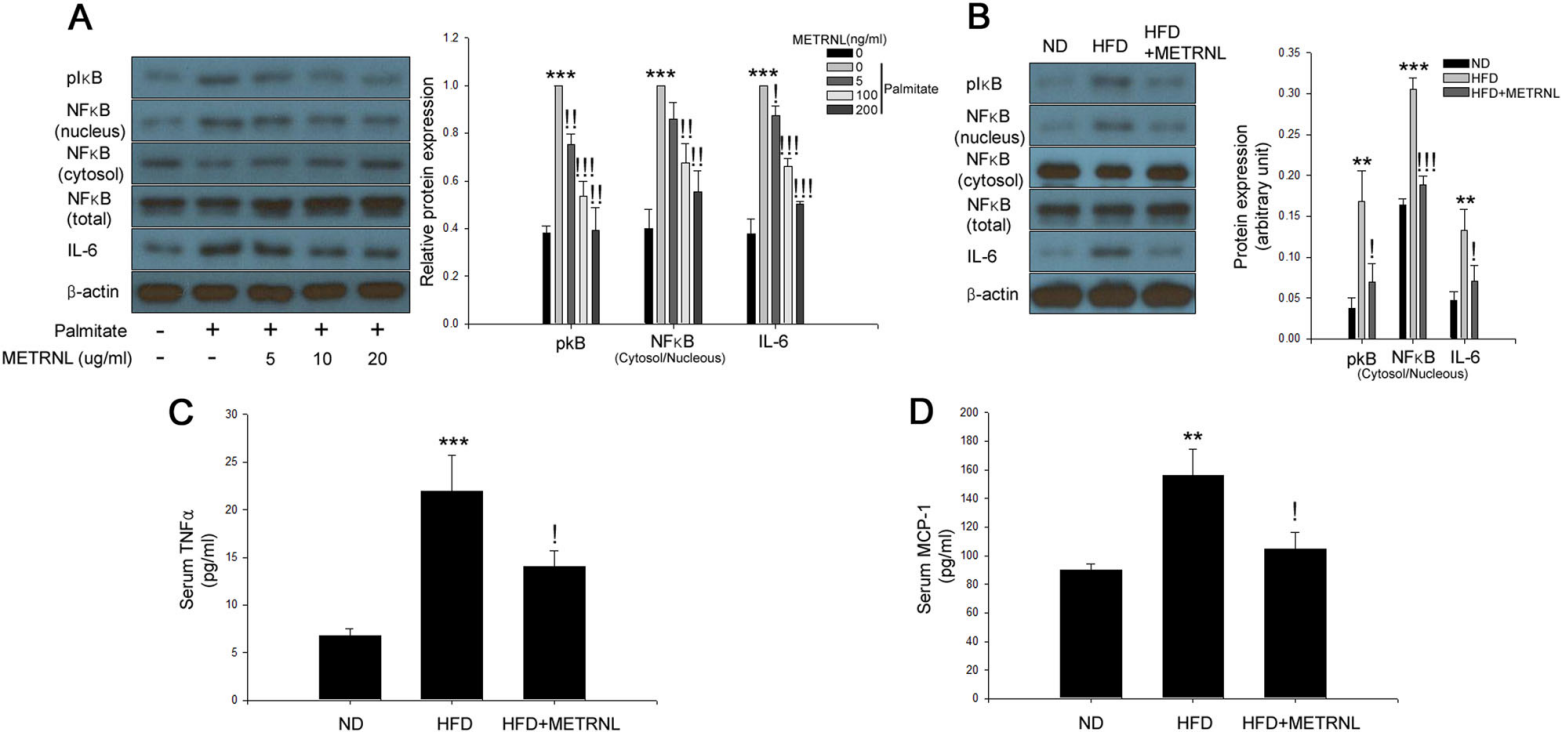
METRNL attenuates inflammation in myotubes. **a** Western blot analysis of palmitate-induced NFκB nuclear translocation and IκB phosphorylation in differentiated C2C12 cells treated with 200 μM palmitate and METRNL (0–200 ng/mL) for 24 h. **b** Western blot analysis of HFD-induced NFκB nuclear translocation and IκB phosphorylation in soleus skeletal muscle of mice treated with METRNL (1 μg/mouse/day) for 8 weeks. Serum analysis of **c** TNFα and **d** MCP-1 of mice treated with HFD and METRNL (5 animals/treatment group). Means ± SEM were calculated from five separate animals. ^***^P < 0.001 and ^**^P < 0.01 compared to control or ND treatment.^!!!^*P* < 0.001,^!!^*P* < 0.01, and ^!^*P* < 0.05 compared to palmitate or HFD treatment

METRNL attenuated palmitate-induced NFκB nuclear translocation, IκBα phosphorylation, and IL-6 expression in a dose-dependent manner (Fig. 3a). Furthermore, treatment with METRNL attenuated the HFD-induced NFκB nuclear translocation and IκBα phosphorylation in mouse skeletal muscle (Fig. 3b). Serum TNFα and monocyte chemotactic factor-1 (MCP-1) were also reduced by METRNL administration (Fig. 3c, d).

### METRNL treatment attenuates hyperlipidemia-induced inflammation, which results in the amelioration of insulin resistance through AMPK/PGC1α-mediated signaling

**Fig. 4:**
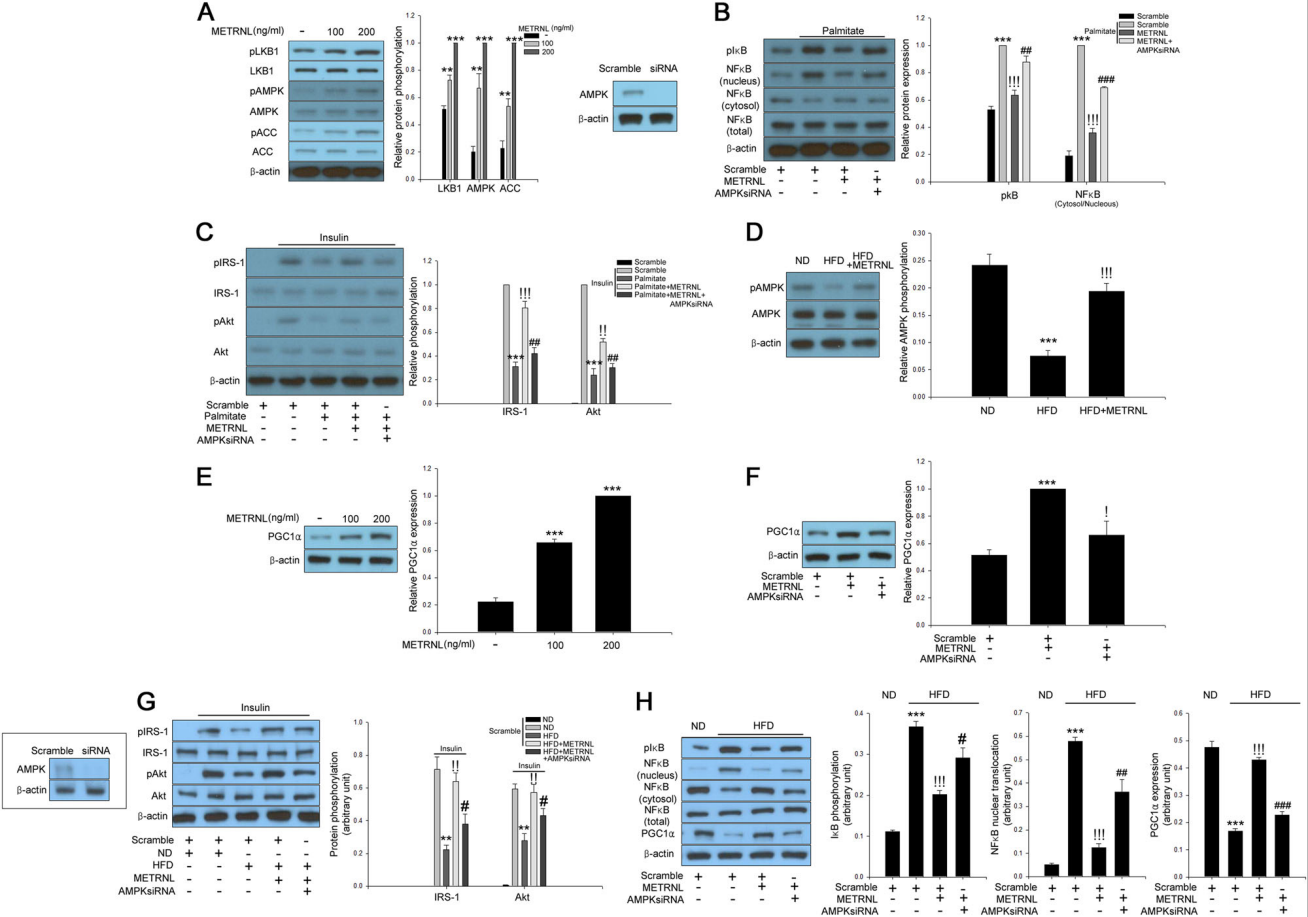
METRNL ameliorates inflammation and insulin resistance via an AMPK-mediated pathway. **a** Western blot analysis of LKB1, AMPK, and ACC phosphorylation in differentiated C2C12 cells treated with METRNL (0–200 ng/mL) for 24 h. **b** Confirmation of AMPK siRNA efficiency in differentiated C2C12 cells. Western blot analysis of palmitate (200 μM)-induced inflammation markers in AMPK siRNA (20 nM)-transfected differentiated C2C12 cells treated with METRNL (0–200 ng/mL) for 24 h. **c** Western blot analysis of the palmitate-induced impairment of IRS-1 and Akt phosphorylation in AMPK siRNA-transfected differentiated C2C12 cells treated with METRNL (0–200 ng/mL) for 24 h. Human insulin (10 nM) was used to stimulate insulin signaling for 3 min. **d** Western blot analysis of AMPK phosphorylation in soleus muscle of mice treated with HFD and METRNL (5 animals/treatment group). **e** Western blot analysis of PGC1α expression in differentiated C2C12 cells treated with METRNL (0–200 ng/mL) for 24 h. **f** Western blot analysis of METRNL (200 ng/mL)-induced PGC1α expression in AMPK siRNA (20 nM)-transfected differentiated C2C12 cells for 24 h. Confirmation of AMPK siRNA efficiency in skeletal muscle of mice. Western blot analysis of IRS-1 and Akt phosphorylation (**g**), and inflammatory markers (**h**) in AMPK siRNA-transfected skeletal muscle of experimental mice. Means ± SEM were obtained from three separate experiments or five animals. ^***^P < 0.001, ^**^P < 0.01, and ^*^P < 0.05 compared to control or ND treatment.^!!!^*P* < 0.001 and ^!!^*P* < 0.01 compared to palmitate or HFD treatment. ^###^*P* < 0.001, ^##^*P* < 0.01, and ^#^*P* < 0.05 compared to palmitate plus METRNL treatment or HFD plus KA group

AMPK has been reported to prevent inflammation^16^. Furthermore, AMPK has been suggested as a therapeutic target for treating insulin resistance and type 2 diabetes^17^. In the present study, the treatment of differentiated C2C12 cells with recombinant METRNL induced phosphorylation of AMPK, as well as ACC, a downstream substrate of AMPK, and LKB1, an upstream AMPK kinase, in a dose-dependent manner (Fig. 4a). We next confirmed the suppressive effects of METRNL on palmitate-induced inflammation through the AMPK-mediated pathway. As shown in Fig. 4b, siRNA-mediated suppression of AMPK mitigated the effects of METRNL on palmitate-induced NFκB nuclear translocation and IκBα phosphorylation (Fig. 4b). We then examined whether METRNL-induced AMPK contributed to the attenuation of insulin resistance caused by palmitate in C2C12 cells. As shown in Fig. 4c, treatment of C2C12 cells with METRNL markedly suppressed palmitate-induced insulin resistance, as detected by an impairment of insulin-stimulated IRS-1 and Akt phosphorylation. In contrast, AMPK siRNA markedly mitigated the effects of METRNL on palmitate-induced insulin resistance (Fig. 4c). METRNL administration markedly reversed the HFD-induced suppression of AMPK phosphorylation in mouse skeletal muscle (Fig. 4d). Exercise-induced PGC1α plays a crucial role in the regulation of muscle metabolism and stimulates the release of METRNL from skeletal muscle^6^. AMPK stimulates PGC1α-mediated signaling^18,19^. PGC1α has previously been shown to exhibit powerful suppressive effects on inflammation^20^ and insulin resistance^21^. Thus, we further examined the effects of METRNL on PGC1α expression. Treatment of C2C12 myocytes with METRNL increased PGC1α expression in a dose-dependent fashion (Fig. 4e). Furthermore, AMPK siRNA abrogated the effects of METRNL on PGC1α expression (Fig. 4f), proving that METRNL induces PGC1α expression through AMPK signaling. To establish the possible links between METRNL, inflammation and insulin resistance more clearly, we performed preliminary animal experiments. To reproduce METRNL-mediated cellular signaling in vitro, experimental mice were treated with HFD and METRNL for 2 weeks. Similar to the in vitro results, suppression of AMPK by in vivo transfection abrogated the effects of METRNL on insulin signaling, inflammation, and PGC1α expression in skeletal muscle of HFD-fed mice (Fig. 4g, h).

### PPARδ/PGC1α signaling plays an important role in the suppressive effects of METRNL on palmitate-induced inflammation and insulin resistance

**Fig. 5:**
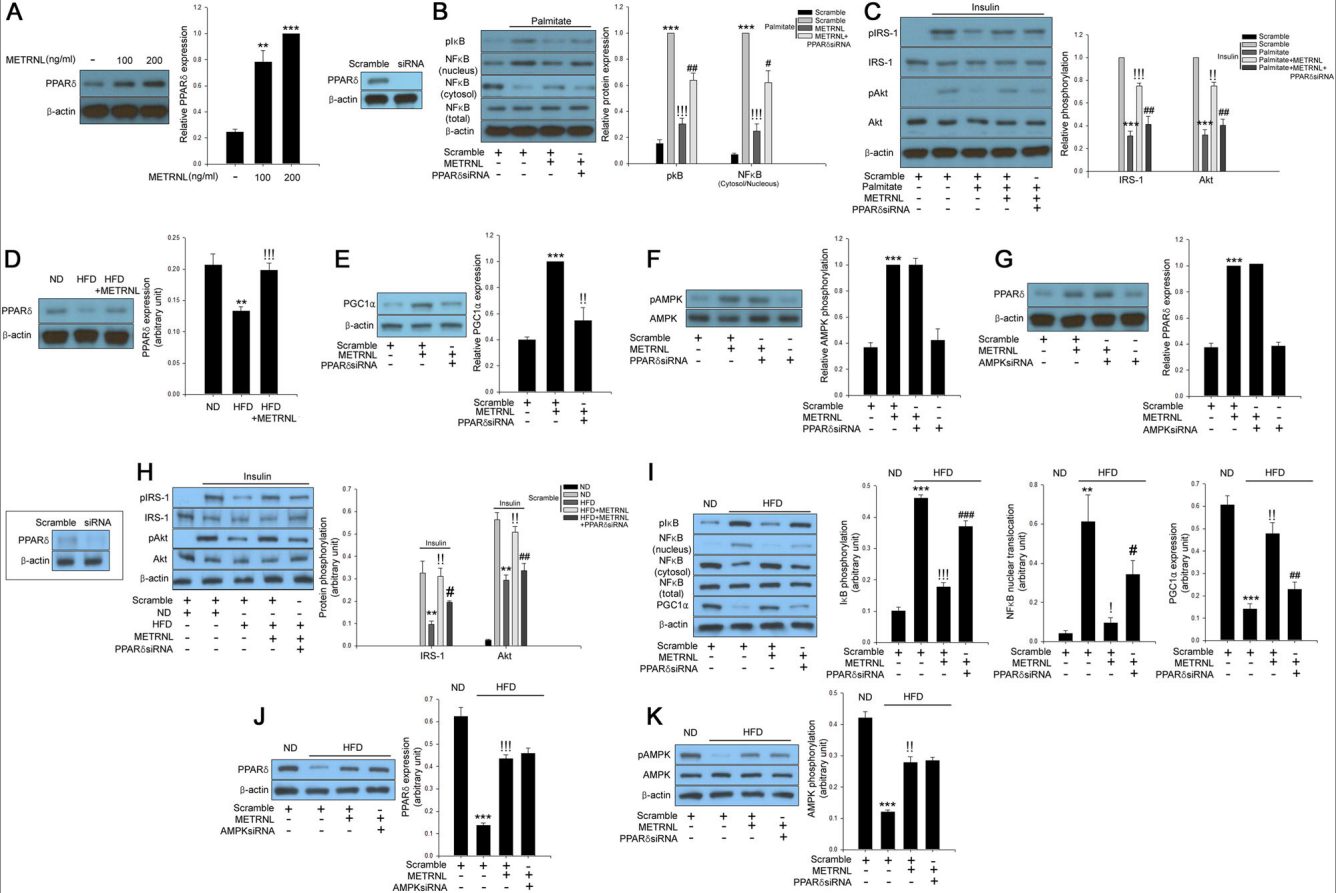
METRNL alleviates inflammation and insulin resistance through a PPARδ-mediated pathway. **a** Western blot analysis of PPARδ expression in differentiated C2C12 cells treated with METRNL (0–200 ng/mL) for 24 h. **b** Confirmation of PPARδ siRNA efficiency in differentiated C2C12 cells. Western blot analysis of palmitate (200 μM)-induced inflammation markers in PPARα siRNA (20 nM)-transfected differentiated C2C12 cells treated with METRNL (0–200 ng/mL) for 24 h. **c** Western blot analysis of the palmitate-induced impairment of IRS-1 and Akt phosphorylation in PPARδ siRNA-transfected differentiated C2C12 cells treated with METRNL (0–200 ng/mL) for 24 h. Human insulin (10 nM) was used to stimulate insulin signaling for 3 min. **d** Western blot analysis of PPARδ expression in soleus muscle of mice treated with HFD and METRNL (5 animals/treatment group). **e** Western blot analysis of METRNL (200 ng/mL)-induced PGC1α expression in PPARδ siRNA (20 nM)-transfected differentiated C2C12 cells for 24 h. Western blot analysis of **f** AMPK phosphorylation in PPARδ siRNA or **g** PPARδ expression in AMPK siRNA-transfected differentiated C2C12 cells treated with METRNL (200 ng/mL) for 24 h. Confirmation of PPARδ siRNA efficiency in skeletal muscle of mice. Western blot analysis of IRS-1 and Akt phosphorylation (**h**), and inflammatory markers (**i**) in PPARδ siRNA-transfected skeletal muscle of experimental mice. **j** Western blot analysis of PPARδ expression in AMPK siRNA-transfected skeletal muscle of experimental mice. **k** Western blot analysis of AMPK phosphorylation in PPARδ siRNA-transfected skeletal muscle of experimental mice. Means ± SEM were obtained from three separate experiments or five animals. ^***^P < 0.001 and ^**^P < 0.01 compared to control or ND treatment. ^!!!^*P* < 0.001, ^!!^*P* < 0.01, and ^!^*P* < 0.05 compared to palmitate, HFD or METRNL treatment. ^###^*P* < 0.001, ^##^*P* < 0.01, and ^#^*P* < 0.05 compared to palmitate plus METRNL treatment

Activation of AMPK has been documented to induce fatty acid oxidation through PPARα and the δ-dependent pathway^22,23^. Furthermore, PPARα, γ, and δ-mediated signaling has been suggested as a target for treating inflammation^24–26^ and insulin resistance^27,28^. Therefore, we investigated whether METRNL can increase PPAR expression in differentiated C2C12 cells. Treatment of C2C12 myocytes with METRNL did not show any significant effects on PPARα or PPARγ expression (Suppl. Figure 2). However, treatment of C2C12 cells with METRNL augmented PPARδ expression in a dose-dependent manner (Fig. 5a). siRNA targeting PPARδ markedly blocked the effects of METRNL on palmitate-induced inflammation and insulin resistance (Fig. 5b, c), demonstrating that METRNL ameliorates palmitate-induced inflammation and insulin resistance through PPARδ-dependent signaling. In agreement with the data from in vitro experiments, METRNL treatment markedly increased the expression of PPARδ in skeletal muscle of HFD-fed mice (Fig. 5d). PPARδ has been reported to activate PGC1α-dependent signaling^29^. siRNA-mediated suppression of PPARδ inhibited METRNL-induced PGC1α expression (Fig. 5e), indicating that PPARδ could contribute to METRNL-induced PGC1α expression. siRNA for PPARδ did not affect METRNL-induced AMPK phosphorylation. Moreover, AMPK siRNA also did not influence METRNL-induced PPARδ expression in C2C12 cells (Fig. 5f, g). Similar to the in vitro results, suppression of PPARδ by in vivo transfection mitigated the effects of METRNL on insulin signaling, inflammation, and PGC1α expression (Fig. 5h, i). Similar to the results seen in (Fig. 5f, g) knockdown of PPARδ did not affect METRNL-induced AMPK phosphorylation (Fig. 5j). Furthermore, AMPK suppression did not influence METRNL-induced PPARδ expression in skeletal muscle of HFD-fed mice (Fig. 5k).

### METRNL stimulates fatty acid oxidation via an AMPK- or PPARδ-dependent pathway in differentiated C2C12 cells and in soleus skeletal muscle of HFD-fed mice

**Fig. 6:**
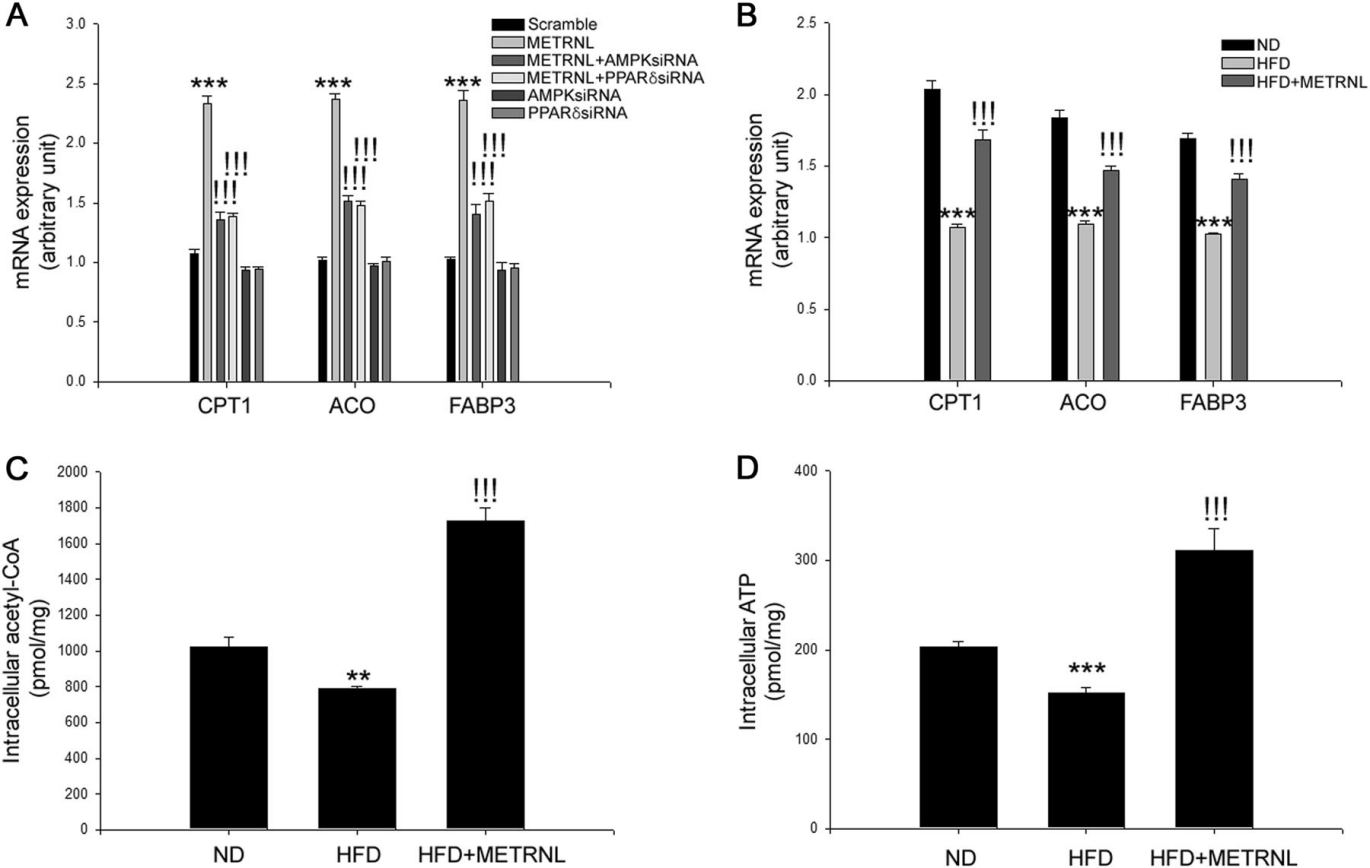
METRNL stimulates fatty acid oxidation-associated gene expression. **a** Quantitative real-time PCR analysis of CPT1, ACO, and FABP3 mRNA expression in AMPK (20 nM) or PPARδ siRNA (20 nM)-transfected C2C12 cells treated with METRNL (200 ng/mL) for 24 h. **b** Quantitative real-time PCR analysis of CPT1, ACO, and FABP3 mRNA expression in HFD-fed mice treated with METRNL. **c** Intracellular acetyl-CoA and **d** ATP levels in soleus skeletal muscle of HFD-fed mice treated with METRNL (1 μg/mouse/day) for 8 weeks (5 animals/treatment group). Means ± SEM were obtained from three separate experiments or five animals. ^***^P < 0.001 and ^**^P < 0.01 compared to control or ND treatment. ^!!!^*P* < 0.001 compared to METRNL or HFD treatment

Koves et al. demonstrated that impaired fatty acid oxidation caused insulin resistance in skeletal muscle^30^. AMPK and PPARδ have been documented to induce fatty acid oxidation^22,31^. Therefore, we evaluated whether METRNL-induced AMPK and PPARδ could enhance fatty acid oxidation in differentiated C2C12 cells. METRNL treatment induced the mRNA expression of fatty acid oxidation-associated genes, such as CPT1, ACO, and FABP3, in C2C12 cells. Suppression of AMPK and PPARδ expression by siRNAs significantly abrogated the inducible effects of METRNL on fatty acid oxidation-associated mRNA expression (Fig. 6a). Moreover, METRNL administration increased CPT1, ACO, and FABP3 mRNA expression in skeletal muscle of mice (Fig. 6b). Fatty acid oxidation products (acetyl-CoA and ATP) were further determined. METRNL markedly increased the levels of intracellular acetyl-CoA and ATP (Fig. 6c, d).

### METRNL did not affect palmitate-induced endoplasmic reticulum (ER) stress in differentiated C2C12 cells

**Fig. 7:**
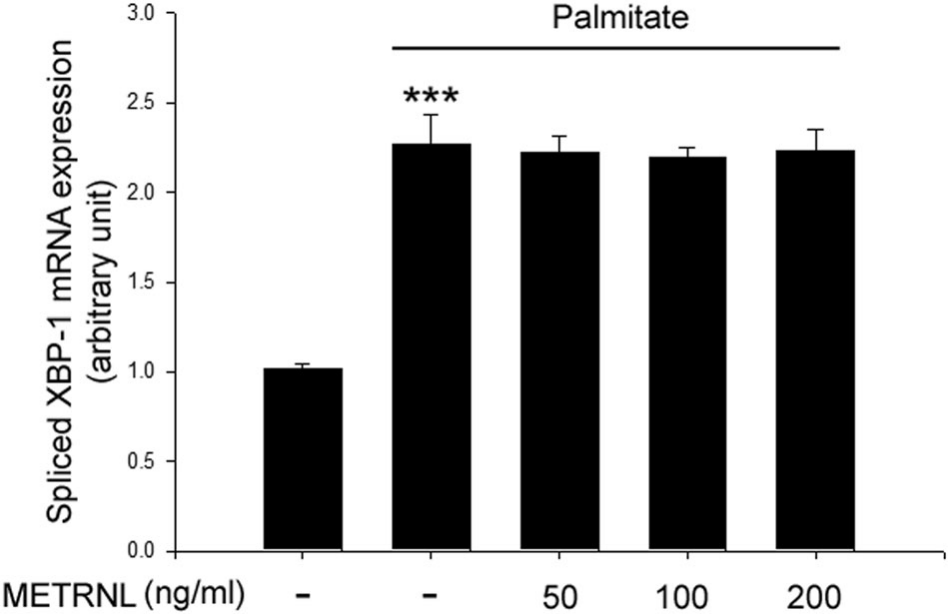
METRNL did not affect palmitate-induced ER stress. Quantitative real-time PCR analysis of spliced XBP-1 mRNA expression in C2C12 cells treated with METRNL (0–200 ng/ml) for 24 h. Means ± SEM were obtained from three separate experiments. ^***^P < 0.001 compared to control

ER stress^32^ has been stated to be is a causative factor in the development of insulin resistance by fatty acids in skeletal muscle. Thus, we next examined whether METRNL suppressed palmitate-induced ER stress. Treatment of differentiated C2C12 myocytes with palmitate markedly increased the expression of spliced XBP-1, an ER stress marker. However, METRNL treatment did not attenuate these palmitate-induced changes (Fig. 7).

## Discussion

Regular physical activity has been demonstrated to have a beneficial effect on insulin sensitivity^1^. During exercise, mRNA expression and release of METRNL from skeletal muscle into the blood are stimulated via PGC1α-mediated signaling activation^6^, which is known to be a key regulator of muscle hypertrophy^33^. METRNL plays an important role in browning white adipose tissue and insulin sensitization via regulation of macrophage activity^5^. Therefore, Rao et al. suggested that METRNL may offer exercise-mediated protection against metabolic disorders^6^. However, the underlying mechanisms by which METRNL may attenuate inflammation and insulin resistance in skeletal muscle remain uncertain.

AMPK plays a pivotal role in cellular energy homeostasis. Additionally, AMPK exerts a significant anti-inflammatory effect via suppression of the NFκB signaling pathway^16^ and immunosuppressive effects^34^. Dysregulation of AMPK plays a critical role in the pathogenesis of insulin resistance and metabolic syndrome-associated diseases in humans as well as in experimental models^35^. Elevated serum free fatty acid levels induced by obesity reduce AMPK activity^36^ and insulin sensitivity^37,38^. Hyperglycemia causes pro-inflammatory actions and oxidative stress^39^. However, activation of AMPK suppresses the production of reactive oxygen species^40^ and mitigates the inflammatory response through induction of thioredoxin. AMPK activation by PPARδ also mitigates ER stress, resulting in attenuation of inflammation and insulin resistance^41^. Increases in AMPK activity were detected in the muscle of patients with type 2 diabetes during physical activity^42^. In the present study, we demonstrated that METRNL markedly augmented AMPK phosphorylation. Additionally, AMPK siRNA blocked the inhibitory effects of METRNL on inflammation and insulin resistance in myotubes. These results suggest and provide insight into one possible mechanism by which the positive effects of physical activity may be associated with METRNL-mediated AMPK signaling.

PPARδ is known to be a critical regulator of the benefits of exercise in various organs, such as the heart, liver, fat, and skeletal muscle. Activation of PPARδ ligand-mediated signaling ameliorates macrophage-associated inflammation and reduces serum triglyceride levels through modulation of lipoprotein metabolism and elevated plasma HDL-cholesterol levels. Activation of the PPARδ pathway also mitigates hepatic gluconeogenesis, resulting in amelioration of hyperglycemia. Furthermore, PPARδ pathway activation attenuates lipopolysaccharide-induced inflammation via suppression of NFκB-mediated signal transduction in cardiomyocytes^24^. Fatty acid oxidation and skeletal muscle energy expenditure by PPARδ bring many positive effects, such as weight loss, stimulation of skeletal muscle metabolic rate, attenuation of insulin resistance and atherogenic inflammation^43^. Thus, PPARδ has emerged as a novel therapeutic target for metabolic disorders^43^. In the present study, we demonstrate for the first time that METRNL treatment can augment PPARδ expression and consequently attenuate the inflammatory response via suppression of NFκB-mediated signaling and pro-inflammatory cytokines.

Herein, METRNL-induced AMPK phosphorylation and PPARδ expression decreased palmitate-induced inflammation. Saturated fatty acids have been previously suggested to cause inflammation through induction of pro-inflammatory cytokines, such as TNFα and IL-6, through NFκB activation^44^. In line with this finding, we reaffirmed that palmitate stimulates NFκB activity, resulting in impairment of insulin signaling, such as IRS-1 and Akt phosphorylation, in differentiated C2C12 cells. AMPK siRNA or PPARδ markedly mitigated the inhibitory effects on palmitate-induced inflammation and insulin resistance, indicating that METRNL-induced AMPK and PPARδ could contribute to the amelioration of inflammation and insulin resistance. In in vivo experiments using HFD-fed mice, METRNL treatment attenuated NFκB-mediated signaling and the levels of pro-inflammatory cytokines (TNFα and MCP-1). Additionally, we found that AMPK and PPARδ are independently regulated by METRNL. These results suggest that METRNL alleviates palmitate- or HFD-induced insulin resistance by ameliorating inflammation via AMPK- or PPARδ-dependent pathways in skeletal muscle. Furthermore, we demonstrated that METRNL increased PGC1α expression through both AMPK- and PPARδ-mediated signaling. These results suggest the possibility that PGC1α may play a central role in the METRNL-mediated attenuation of inflammation and insulin resistance in skeletal muscle. However, further studies are needed to elucidate this assumption.

The present study showed that METRNL administration improved both glucose tolerance and insulin tolerance in experimental mice. Elevated basal glucose levels were observed in HFD-fed mice during IPGTTs and ITTs. Therefore, the percentage of basal glucose levels was graphed to determine insulin sensitivity. Moreover, we demonstrated that METRNL administration suppressed basal glucose levels. These results may be associated with hepatic gluconeogenesis or glucose uptake by various organs through AMPK^45,46^, PPARδ^47,48^, or other pathways.

In this study, METRNL treatment reduced the body weight of mice but did not influence calorie intake. These results suggest that body weight loss may be caused by fatty acid oxidation-mediated fat burning in adipose tissue and browning of white adipose tissue through the PGC1α-mediated pathway^6^, as METRNL-mediated internal mechanisms. However, no changes have been observed in body weight or energy expenditure in either METRNL transgenic mice or knockout mice^7^. This lack of an effect may be due to differences in experimental animal models and related conditions. Therefore, further studies are needed to investigate the effects of METRNL on fatty acid metabolism in adipose tissue. Additionally, METRNL did not attenuate palmitate-induced ER stress, which has been reported to play a causal role in the development of insulin resistance in skeletal muscle^32^, disclosing that the protective effects of METRNL on inflammation and insulin resistance are not related to ER stress.

**Fig. 8:**
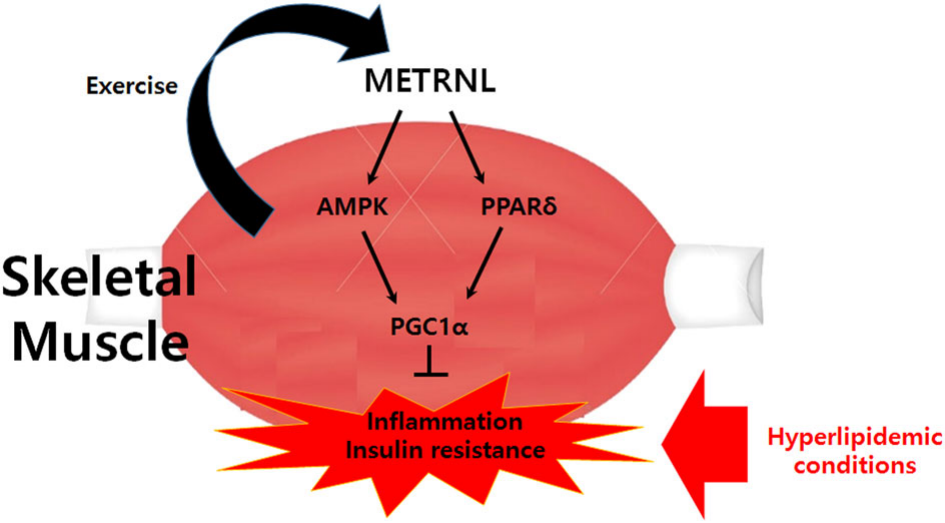
Schematic diagram showing the effects of METRNL on skeletal muscle insulin resistance

In conclusion, the current study is the first to show that METRNL ameliorates lipid-induced inflammation and insulin resistance via AMPK- or PPARδ-dependent signaling in skeletal muscle of mice (Fig. 8). These results may shed light on the novel mechanisms associated with the positive effects of physical activity and suggest an attractive therapeutic strategy for treating metabolic syndrome, including insulin resistance.

## Electronic supplementary material

Supplemental Figure 1

Supplemental Figure 2

Supplementary Figure Legends

## References

[1] Bogardus C (1984). Effects of physical training and diet therapy on carbohydrate metabolism in patients with glucose intolerance and non-insulin-dependent diabetes mellitus. Diabetes.

[2] Schnyder S, Handschin C (2015). Skeletal muscle as an endocrine organ: PGC-1alpha, myokines and exercise. Bone.

[3] Li ZY (2014). Subfatin is a novel adipokine and unlike Meteorin in adipose and brain expression. Cns. Neurosci. Ther..

[4] Ushach I (2015). METEORIN-LIKE is a cytokine associated with barrier tissues and alternatively activated macrophages. Clin. Immunol..

[5] Zheng SL, Li ZY, Song J, Liu JM, Miao CY (2016). Metrnl: a secreted protein with new emerging functions. Acta Pharmacol. Sin..

[6] Rao RR (2014). Meteorin-like is a hormone that regulates immune-adipose interactions to increase beige fat thermogenesis. Cell.

[7] Li ZY (2015). Adipocyte metrnl antagonizes insulin resistance through PPARgamma signaling. Diabetes.

[8] Boden G (1997). Role of fatty acids in the pathogenesis of insulin resistance and NIDDM. Diabetes.

[9] Vessby B (2001). Substituting dietary saturated for monounsaturated fat impairs insulin sensitivity in healthy men and women: the KANWU Study. Diabetologia.

[10] Coll T (2008). Oleate reverses palmitate-induced insulin resistance and inflammation in skeletal muscle cells. J. Biol. Chem..

[11] Senn JJ (2006). Toll-like receptor-2 is essential for the development of palmitate-induced insulin resistance in myotubes. J. Biol. Chem..

[12] Shi H (2006). TLR4 links innate immunity and fatty acid-induced insulin resistance. J. Clin. Invest..

[13] Kim JK (2001). Prevention of fat-induced insulin resistance by salicylate. J. Clin. Invest..

[14] Kern PA, Ranganathan S, Li C, Wood L, Ranganathan G (2001). Adipose tissue tumor necrosis factor and interleukin-6 expression in human obesity and insulin resistance. Am. J. Physiol. Endocrinol. Metab..

[15] Bae JY (2018). Aerobic exercise increases meteorin-like protein in muscle and adipose tissue of chronic high-fat diet-induced obese mice. Biomed. Res. Int..

[16] Salminen A, Hyttinen JM, Kaarniranta K (2011). AMP-activated protein kinase inhibits NF-kappaB signaling and inflammation: impact on healthspan and lifespan. J. Mol. Med (Berl.).

[17] Zhang B B, Zhou G, Li C (2009). AMPK: an emerging drug target for diabetes and the metabolic syndrome. Cell. Metab..

[18] Qin ZY (2014). Metformin prevents LYRM1-induced insulin resistance in 3T3-L1 adipocytes via a mitochondrial-dependent mechanism. Exp. Biol. Med..

[19] Jager S, Handschin C, St-Pierre J, Spiegelman BM (2007). AMP-activated protein kinase (AMPK) action in skeletal muscle via direct phosphorylation of PGC-1alpha. Proc. Natl Acad. Sci. USA.

[20] Handschin C, Spiegelman BM (2008). The role of exercise and PGC1alpha in inflammation and chronic disease. Nature.

[21] Kleiner S (2012). Development of insulin resistance in mice lacking PGC-1alpha in adipose tissues. Proc. Natl Acad. Sci. USA.

[22] Lee WJ (2006). AMPK activation increases fatty acid oxidation in skeletal muscle by activating PPARalpha and PGC-1. Biochem. Biophys. Res. Commun..

[23] Narkar VA (2008). AMPK and PPARdelta agonists are exercise mimetics. Cell.

[24] Ding G, Cheng L, Qin Q, Frontin S, Yang Q (2006). PPARdelta modulates lipopolysaccharide-induced TNFalpha inflammation signaling in cultured cardiomyocytes. J. Mol. Cell. Cardiol..

[25] Kilgore KS, Billin AN (2008). PPARbeta/delta ligands as modulators of the inflammatory response. Curr. Opin. Investig. Drugs.

[26] Clark RB (2002). The role of PPARs in inflammation and immunity. J. Leukoc. Biol..

[27] Lee CH (2006). PPARdelta regulates glucose metabolism and insulin sensitivity. Proc. Natl Acad. Sci. USA.

[28] Barish GD, Narkar VA, Evans RM (2006). PPAR delta: a dagger in the heart of the metabolic syndrome. J. Clin. Invest..

[29] Hondares, E.et al. PPARdelta, but not PPARalpha, activates PGC-1alpha gene transcription in muscle. *Biochem. Biophys. Res. Commun.* **354**, 1021–1027 (2007) . 10.1016/j.bbrc.2007.01.092 17275789

[30] Koves TR (2008). Mitochondrial overload and incomplete fatty acid oxidation contribute to skeletal muscle insulin resistance. Cell. Metab..

[31] Tanaka T (2003). Activation of peroxisome proliferator-activated receptor delta induces fatty acid beta-oxidation in skeletal muscle and attenuates metabolic syndrome. Proc. Natl Acad. Sci. USA.

[32] Peng G (2011). Oleate blocks palmitate-induced abnormal lipid distribution, endoplasmic reticulum expansion and stress, and insulin resistance in skeletal muscle. Endocrinology.

[33] Ruas JL (2012). A PGC-1alpha isoform induced by resistance training regulates skeletal muscle hypertrophy. Cell.

[34] Salt IP, Palmer TM (2012). Exploiting the anti-inflammatory effects of AMP-activated protein kinase activation. Expert. Opin. Investig. Drugs.

[35] Ruderman Neil B., Carling David, Prentki Marc, Cacicedo José M. (2013). AMPK, insulin resistance, and the metabolic syndrome. Journal of Clinical Investigation.

[36] Wu Y, Song P, Xu J, Zhang M, Zou MH (2007). Activation of protein phosphatase 2A by palmitate inhibits AMP-activated protein kinase. J. Biol. Chem..

[37] Boden G (2003). Effects of free fatty acids (FFA) on glucose metabolism: significance for insulin resistance and type 2 diabetes. Exp. Clin. Endocrinol. Diabetes.

[38] Boden G (1999). Free fatty acids, insulin resistance, and type 2 diabetes mellitus. Proc. Assoc. Am. Physicians.

[39] de Carvalho Vidigal F, Guedes Cocate P, Goncalves Pereira L, de Cassia, Goncalves Alfenas R (2012). The role of hyperglycemia in the induction of oxidative stress and inflammatory process. Nutr. Hosp..

[40] Cardaci S, Filomeni G, Ciriolo MR (2012). Redox implications of AMPK-mediated signal transduction beyond energetic clues. J. Cell. Sci..

[41] Salvado L (2014). PPARbeta/delta prevents endoplasmic reticulum stress-associated inflammation and insulin resistance in skeletal muscle cells through an AMPK-dependent mechanism. Diabetologia.

[42] Musi N (2001). AMP-activated protein kinase (AMPK) is activated in muscle of subjects with type 2 diabetes during exercise. Diabetes.

[43] Reilly SM, Lee CH (2008). PPAR delta as a therapeutic target in metabolic disease. FEBS Lett..

[44] Tripathy D (2003). Elevation of free fatty acids induces inflammation and impairs vascular reactivity in healthy subjects. Diabetes.

[45] Mihaylova MM, Shaw RJ (2011). The AMPK signalling pathway coordinates cell growth, autophagy and metabolism. Nat. Cell Biol..

[46] Musi N, Goodyear LJ (2003). AMP-activated protein kinase and muscle glucose uptake. Acta Physiol. Scand..

[47] Souza-Mello V (2015). Peroxisome proliferator-activated receptors as targets to treat non-alcoholic fatty liver disease. World J. Hepatol..

[48] Kramer DK (2005). Direct activation of glucose transport in primary human myotubes after activation of peroxisome proliferator-activated receptor delta. Diabetes.

