# Supplementary figures and images for: METRNL attenuates lipid-induced inflammation and insulin resistance via AMPK or PPARδ-dependent pathways in skeletal muscle of mice
Source: Exp Mol Med. 2018 Sep 13;50(9):122. doi: 10.1038/s12276-018-0147-5 (PMC6137187; doi:10.1038/s12276-018-0147-5)

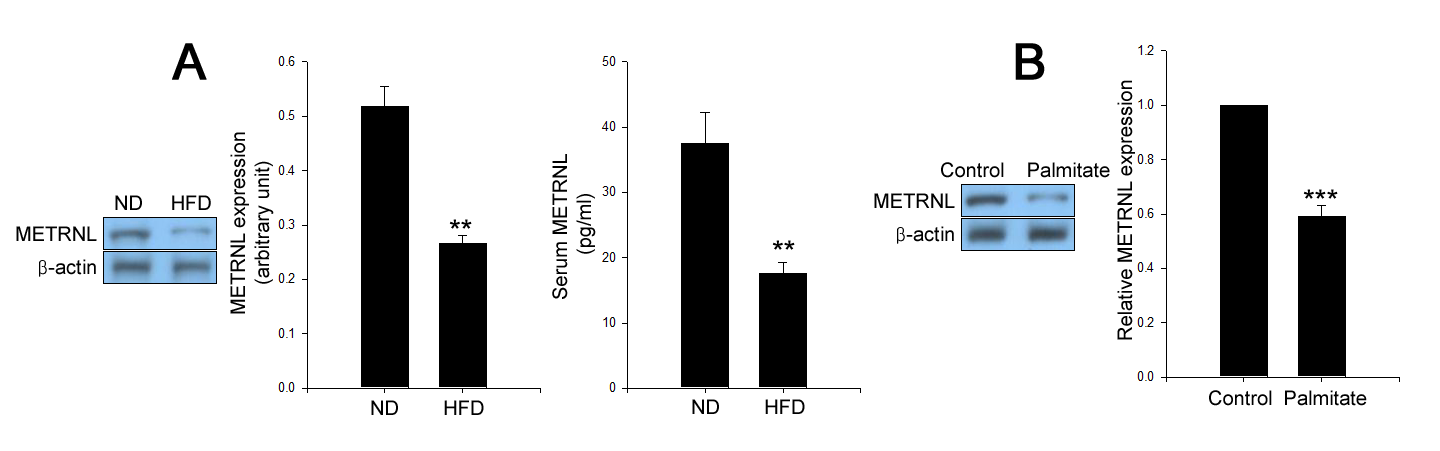

Supplement: Supplementary file 1 — Supplemental Figure 1 [file 12276_2018_147_MOESM1_ESM.tif]

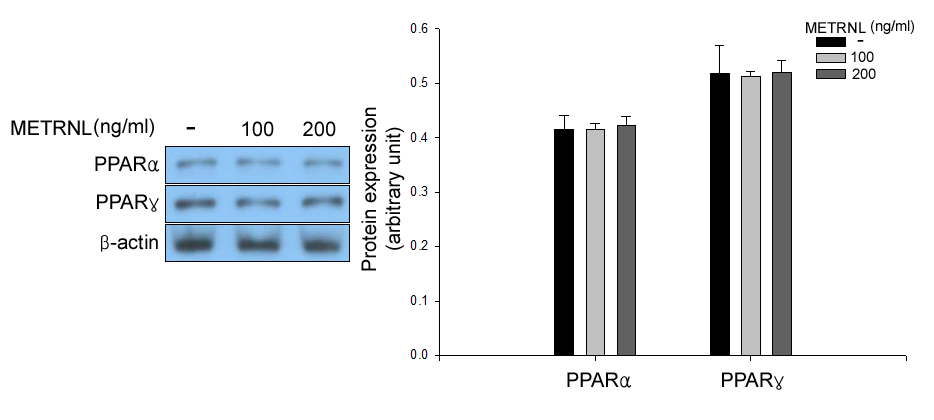

Supplement: Supplementary file 2 — Supplemental Figure 2 [file 12276_2018_147_MOESM2_ESM.tif]
