# Supplementary material for: METRNL attenuates lipid-induced inflammation and insulin resistance via AMPK or PPARδ-dependent pathways in skeletal muscle of mice
Source: Exp Mol Med. 2018 Sep 13;50(9):122. doi: 10.1038/s12276-018-0147-5 (PMC6137187; doi:10.1038/s12276-018-0147-5)
Supplement: Supplementary file 3 — Supplementary Figure Legends [file 12276_2018_147_MOESM3_ESM.docx]

Suppl. Fig. 1 **Downregulation of** **METRNL in skeletal muscle under hyperlipidemic conditions.** (A) Western blot analysis of METRNL expression in soleus muscle of mice treated with HFD. Serum analysis of METRNL in HFD-fed mice (3 animals/treatment group). (B) Western blot analysis of METRNL expression in C2C12 myocytes treated with palmitate. Means ± SEM were calculated from five separate animals. ^***^*P*<0.001 and ^**^*P*<0.01 compared to control or ND treatment.

Suppl. Fig. 2 **METRNL did not affect PPARα or PPARγ expression in C2C12 myocytes.** Western blot analysis of PPARα and PPARγ expression in C2C12 myocytes treated with METRNL (0-200 ng/mL) for 24 h.
